# Rabies Exposures, Post-Exposure Prophylaxis and Deaths in a Region of Endemic Canine Rabies

**DOI:** 10.1371/journal.pntd.0000339

**Published:** 2008-11-25

**Authors:** Katie Hampson, Andy Dobson, Magai Kaare, Jonathan Dushoff, Matthias Magoto, Emmanuel Sindoya, Sarah Cleaveland

**Affiliations:** 1 Department of Animal and Plant Sciences, University of Sheffield, Western Bank, Sheffield, United Kingdom; 2 Department of Ecology and Evolutionary Biology, Princeton University, Princeton, New Jersey, United States of America; 3 Centre for Infectious Diseases, School of Biological Sciences, University of Edinburgh, King's Buildings, Ashworth Laboratories, Edinburgh, Midlothian, United Kingdom; 4 Department of Biology, McMaster University, Hamilton, Ontario, Canada; 5 Minstry of Water and Livestock Development, Serengeti District Livestock Office, Mugumu, Tanzania; 6 Division of Infection and Immunity, The Roslin Institute/Royal (Dick) School of Veterinary Studies, University of Edinburgh, Easter Bush, Roslin, Midlothian, United Kingdom; WHO-IVR, Switzerland

## Abstract

**Background:**

Thousands of human deaths from rabies occur annually despite the availability of effective vaccines following exposure, and for disease control in the animal reservoir. Our aim was to assess risk factors associated with exposure and to determine why human deaths from endemic canine rabies still occur.

**Methods and Findings:**

Contact tracing was used to gather data on rabies exposures, post-exposure prophylaxis (PEP) delivered and deaths in two rural districts in northwestern Tanzania from 2002 to 2006. Data on risk factors and the propensity to seek and complete courses of PEP was collected using questionnaires. Exposures varied from 6–141/100,000 per year. Risk of exposure to rabies was greater in an area with agropastoralist communities (and larger domestic dog populations) than an area with pastoralist communities. Children were at greater risk than adults of being exposed to rabies and of developing clinical signs. PEP dramatically reduced the risk of developing rabies (odds ratio [OR] 17.33, 95% confidence interval [CI] 6.39–60.83) and when PEP was not delivered the risks were higher in the pastoralist than the agro-pastoralist area (OR 6.12, 95% CI 2.60–14.58). Low socioeconomic class and distance to medical facilities lengthened delays before PEP delivery. Over 20% of rabies-exposed individuals did not seek medical treatment and were not documented in official records and <65% received PEP. Animal bite injury records were an accurate indicator of rabies exposure incidence.

**Conclusions:**

Insufficient knowledge about rabies dangers and prevention, particularly prompt PEP, but also wound management, was the main cause of rabies deaths. Education, particularly in poor and marginalized communities, but also for medical and veterinary workers, would prevent future deaths.

## Author Summary

Thousands of human deaths from rabies occur annually despite availability of effective vaccines for humans following exposure, and for disease control in domestic dog populations. We established a 5-year contact-tracing study in northwest Tanzania to investigate risk factors associated with rabies exposure and to determine why human deaths from canine rabies still occur. We found that children were at greater risk of being bitten and of developing rabies than adults and that incidence of bites by suspected rabid animals was higher in an area with larger domestic dog populations. A large proportion (>20%) of those bitten by rabid animals are not recorded in official records because they do not seek post-exposure prophylaxis (PEP), which is crucial for preventing the onset of rabies. Of those that seek medical attention, a significant proportion do not receive PEP because of the expense or because of hospital shortages; and victims who are poorer, and who live further from medical facilities, typically experience greater delays before obtaining PEP. Our work highlights the need to raise awareness about rabies dangers and prevention, particularly prompt PEP, but also wound management. We outline practical recommendations to prevent future deaths, stressing the importance of education, particularly in poor and marginalized communities, as well as for medical and veterinary workers.

## Introduction

Rabies is an acute viral infection which causes horrifying neurological symptoms that inevitably result in death. Although human rabies encephalitis remains untreatable [1], the infection is entirely preventable, both by post-exposure prophylaxis (PEP) of bite victims, and by population-level vaccination of the zoonotic reservoir, which across most of Africa and Asia is the domestic dog [2]. Modern cell culture vaccines used in combination with rabies immunoglobulins are virtually 100% effective in preventing human deaths if administered promptly to rabies-exposed patients following appropriate wound management [3] and mass vaccination of domestic dogs has successfully eliminated or controlled domestic dog rabies in many parts of the world [4],[5]. It is therefore inexcusable that an estimated 55,000 human deaths from rabies occur annually [6], of which over 99% are in developing countries where the disease is endemic in domestic dog populations [7].

Recent estimates of human rabies mortality are based upon a probability decision-tree model [6], because current surveillance systems have been shown to substantially underreport the number of deaths from rabies. For example, in Tanzania more than 100 human rabies deaths are estimated to occur for each officially reported case [6]. Hospital studies further suggest that clinical diagnosis of human rabies may be hindered by confusion with common neurological syndromes, such as cerebral malaria [8]. These and other studies on rabies incidence and exposure risk rely on bite victims reporting to hospital, yet not all rabies-exposed individuals seek medical attention. To investigate the validity of methods being used to estimate the burden of rabies we established a contact-tracing study. Data collected using these methods provides a more comprehensive picture of the reality facing communities in regions where canine rabies is endemic. Using these data we quantify the risk of disease and exposure and attempt to understand why human deaths from canine rabies still occur and thus how this number can be reduced.

## Methods

### Contact-tracing

Data was collected from two rural districts in northwest Tanzania: Serengeti, which is inhabited by multi-ethnic, agro-pastoralist communities and high-density dog populations, and Ngorongoro, which is inhabited by low-density pastoralist communities and lower density dog populations. Contact tracing of potential rabies-exposures was initiated using data from hospitals and medical dispensaries on patients with animal-bite injuries, and case reports from livestock offices and community-based surveillance activities. Visits were made to investigate incidents that occurred between January 2002 and December 2006 involving potentially suspect rabid animals. Interviews were conducted to assess the case history and identify the source of exposure and other contacts if known. The same procedure was followed for all resulting exposures and preceding cases where identified, and UTM coordinates were recorded at each household and at the location of the exposure event (where possible). Interviews were conducted by veterinary or livestock field-officers, often with a community leader in attendance. This created an active local reporting network. Animal cases were diagnosed on epidemiological and clinical criteria adapting the ‘six-step’ method through retrospective interviews with witnesses [9]. Wherever possible brain samples from animals that caused bite injuries were collected and tested for case confirmation [10].

### Questionnaires

A structured open-ended questionnaire was administered to bite victims at 3 designated district hospitals (in Magu, Misungwi and Tarime, n = 166) to obtain information on intervals between exposure and reporting to hospital for PEP, and ways used to raise funds to pay for PEP. Information was collected on household socioeconomic status, using indicators sensitive to local determinants of wealth, previously identified through Rapid Rural Appraisal approaches [11]. Specifically numbers of cattle and housing quality were chosen as independent wealth indicators because individuals may own many cattle and hence be considered to be wealthy but they may not necessarily own “modern” houses. Individuals with houses constructed from cement/baked bricks, which have cement floors and corrugated roofs were categorized as belonging to high socioeconomic status and those owning houses constructed from other materials were classified as low socioeconomic status. Regardless of housing quality, individuals owning >50 heads of cattle were categorized as high socioeconomic status; those with <50 heads were classified as low socioeconomic status. UTM coordinates were collected for each district hospital and household visited.

The study was approved by the Tanzania Commission for Science and Technology with ethical review from the National Institute for Medical Research (NIMR). In Tanzania, NIMR ethical guidelines stipulate that written consent is required for participants in clinical trials. However, as this was a retrospective study involving collection of interview data only, without clinical intervention or sampling, we considered that informed verbal consent was appropriate and this was approved by NIMR. Permission to conduct interviews was obtained from district officials, village and sub-village leaders in all study locations. At each household visited, the head of the household was informed about the purpose of the study and interviews were only subsequently conducted following verbal consent from both the head of the household and the bite victim.

### Statistical Analysis

Bite-injury records were compiled for hospitals in Serengeti and Ngorongoro districts and neighboring districts of Tarime, Musoma and Bunda. Records were extracted for patients originating from Serengeti and Ngorongoro and correlations with rabies exposures and observations of rabid animals were examined by regression. Fisher's Exact Test was used to determine whether any factors were associated with delays in PEP delivery and to assess differences in the source of funds used to pay for PEP by different socioeconomic classes. Binomial confidence intervals were reported for proportions. Chi-square tests were used to examine differences in exposure incidence across age-classes, and to different parts of the body. The odds of developing rabies following exposure and associated risk factors were calculated by logistic regression. All statistical analyses were implemented with the statistical programming language R.

## Results

### Exposures

1080 people were traced and interviewed who had been bitten by animals between 2002 and 2006 in Serengeti (776) and Ngorongoro (304) districts. On the basis of descriptive case histories >97% of animals that caused bite injuries were classified as suspected rabid (648) or normal (406). The status of animals that bit the remaining 2.5 percent (26) of cases visited was unclear. Approximately 75% of samples from suspected rabid animals tested positive, indicating that recognition of rabies is accurate and that classification using the case history description is valid [12]. Over twenty-five percent of visited cases bitten by suspected rabid animals (180) were identified through contact tracing alone because the victim did not seek medical attention. Of 1322 bite injury records from medical facilities over the same period, 57% (760) were successfully traced, 9% (118) were not visited because the record indicated the animal was healthy and the remaining 444 cases were either impossible to trace, not present to interview, or have yet to be visited (139 were from 2006). At least 50 of these exposures were by suspected rabid animals.

Conservative estimates suggest around 63/100,000 people in Serengeti and 17/100,000 in Ngorongoro are bitten by suspected rabid animals annually. Including animals of undetermined status raises those figures to 100 and 30 exposures/100,000 respectively. The risk of being bitten by a suspected rabid animal varied through time (approaching 150/100,000 during the epidemic peak), but was consistently higher in Serengeti, the more populated district (Table 1). Most suspected rabies exposures were due to domestic animals (89%), particularly dogs (Table 2). A higher proportion of bites by suspected rabid animals were from wild animals in Ngorongoro district compared to Serengeti district (∼20% versus <10%), but annual incidence of bites by wild animals was still lower in Ngorongoro than Serengeti (0.5 versus 0.7/100,000). The seventy-one exposures by suspected rabid wild animals were predominantly due to jackals (23), hyenas (20) and honey badgers (17), with additional exposures from white-tailed mongooses (5), bat-eared foxes (2), genets (2), wildcats (2) and a leopard (2). 75% of victims bitten by suspected rabid hyenas required prolonged hospital stays due to the severity of their injuries.

**Table 1:** Incidence of rabies exposures and deaths and the probability of developing rabies following exposure in Serengeti and Ngorongoro Districts.

	Bites/ 100,000				Deaths/ 100,000	
	Serengeti District		Ngorongoro District		Serengeti District	Ngorongoro District
Year	lower	Upper	lower	upper		
2002	17.78	24.09	8.14	18.50	1.15	1.48
2003	111.80	135.84	43.48	54.18	1.12	4.28
2004	94.80	140.57	19.23	37.77	2.72	2.06
2005	49.92	90.28	7.94	29.11	1.06	1.32
2006	40.89	108.18	5.74	11.47	1.55	0.64
**Average**	63.04	99.79	16.91	30.21	1.51	2.29
**Probability of developing rabies following exposure**					0.02	0.12

Lower estimates are based on successfully traced exposures determined to be from suspected rabid animals and records that indicated the bite was caused by an animal suspected to be rabid. Upper estimates include bite injuries where the status of the biting animal was not recorded and the case has not been traced.

**Table 2:** Suspected rabies exposures by different species in Serengeti and Ngorongoro districts.

Species	Serengeti District (%)	Ngorongoro District (%)
domestic dogs	487 (85.6)	84 (70.1)
domestic cats	26 (4.6)	6 (5.0)
Livestock	7 (1.2)	3 (2.5)
Human	2 (0.4)	1 (0.8)
Wildlife	47 (8.3)	26 (21.7)
**TOTAL**	569	120

Numbers (and percentages) of exposures are shown.

**Figure 1:**
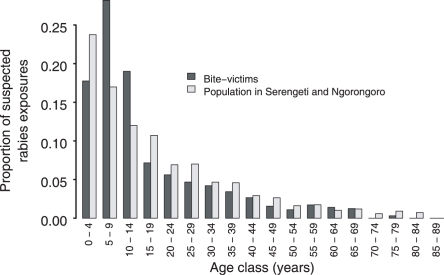
Age distribution of rabies-exposed individuals. The age distribution of suspected rabies bite victims (dark gray, n = 642) compared to the population as a whole in Serengeti and Ngorongoro districts (light gray, n = 307,099).

Children were most at risk of exposure to rabies: 65% of exposures were children (<16 yrs, median 12, range 1–79); children from 5–15 years old had an elevated risk of exposure compared to the rest of the population (Fig. 1, p<0.001); and a higher probability of being bitten on the head, face, or neck (Table 3, p = 0.008). The ratio of male to female exposures was 0.52∶0.48.

**Table 3:** Rabies exposures and deaths according to bite site and age of victim.

Age	Arm	Head	Leg	Trunk	Total
0–10 yrs	53 (2)	23 (6)	55 (2)	24 (1)	155 (11)
10–20 yrs	50 (4)	8 (0)	64 (3)	13 (0)	135 (7)
20+ yrs	41 (2)	9 (0)	64 (1)	8 (0)	122 (3)
**Total**	144 (8)	40 (6)	183 (6)	45 (1)	412 (21)

Numbers in parentheses are rabies deaths. When the victim was bitten multiple times, the bite site closest to the head was listed.

**Figure 2:**
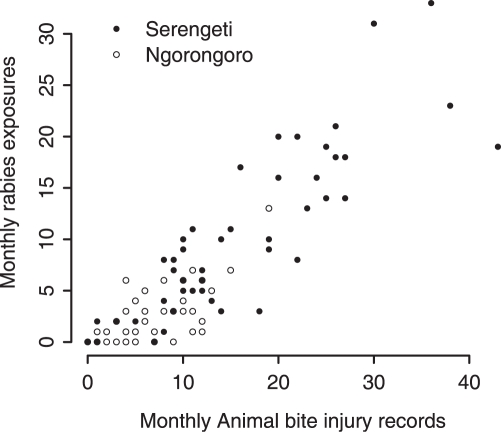
Correlation between the monthly number of exposures by suspected rabid animals and the corresponding number of animal-bite injury records during that month. Records of patients from Serengeti and Ngorongoro districts.

Animal-bite injury records were correlated with suspected rabies exposures in both districts (Fig. 2, p<0.0001, excluding 2006 data because of incomplete contact tracing), although less variation was explained in Ngorongoro (r^2^ = 50%) than Serengeti district (r^2^ = 74%). Some rabies-exposed patients were recorded in hospitals of neighboring districts, not their district hospital, particularly during periods of vaccine shortage. Bite injury records were also correlated with monthly numbers of reported rabid animals (p<0.001), although the relationship was weaker (r^2^ = 58% in Serengeti and 48% in Ngorongoro) due largely to variation in biting behavior of individual rabid animals.

### Treatments

Between 15 and 24% of suspected rabies exposures (169 people, Table 4) did not seek medical attention and so did not receive prompt PEP, though some may have subsequently attended a hospital as a result of the study (advice on rabies dangers and prevention was given at every household visited, including accessible sources of PEP and although we did not provide PEP we occasionally transported exposed bite victims to medical facilities). More than 10% of suspected rabies exposures that attended a medical facility did not receive PEP because none was available (nor was sought or found elsewhere), because the patient was unable to pay, or because of inappropriate medical advice. Overall, only 65% of identified rabies exposures received PEP.

**Table 4:** Numbers of rabies-exposed individuals who attended hospital and received PEP.

	Exposed	Attended hospital	Received PEP
Traced cases	699	530 (76%)	456 (86%)
Traced cases and cases of unknown status	1140	971 (85%)	685 (71%)

The lower row also shows individuals bitten by animals of unknown status who have yet to be traced.

The cost of PEP and the regimen delivered varied depending upon the health facility and the date of presentation, varying from >100,000 Tsh (∼US$85) to free (for limited periods), although courses were typically 75,000 Tsh in Ngorongoro district (five doses) and 30,000 in Serengeti (3 doses), in comparison to monthly per capita expenditure and per household expenditure of 8,538 Tsh and 52,649 Tsh respectively in 2001 [11] (although in 2008 prices are now approaching ∼30,000 Tsh per dose). However, the probability of receiving PEP following exposure was very similar in the two districts (0.70 in Serengeti versus 0.68 in Ngorongoro). Rabies immunoglobulins were not offered to any bite victims.

**Figure 3:**
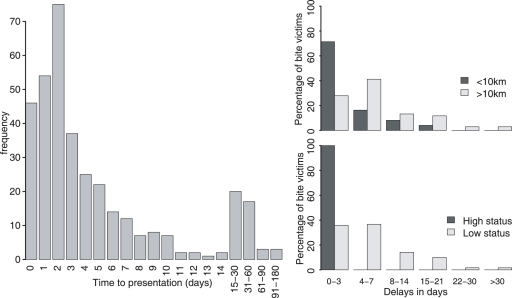
Factors affecting delays to delivery of PEP following exposure. (A) Distribution of delays till first dose of PEP. (B) Delivery delays by distance from district hospital and (C) by socioeconomic status.

Most people who attended a medical facility did so shortly after exposure, but there was considerable variance in delays before receiving the first dose of PEP (Fig. 3); at least 25% of courses were started more than one week later. Distance from the nearest medical facility and socioeconomic status were both significant predictors of delays in PEP delivery (p<0.0001 in both cases, Fig. 3). Of victims that attended hospital for PEP, those located near district hospitals (<10 km) reported earlier than those located further away, with 85.7% (95% CI 77–92%) of victims near district hospitals reporting within 7 days of exposure compared to only 66.2% (54–76%) of victims located farther away. Bite victims of high socioeconomic status reported significantly earlier to hospital than those of low status (p<0.0001). All bite victims with high socioeconomic status that reported to a medical facility did so within three days of being bitten compared with only 24% (95% CI 17–33%) of victims with low socioeconomic status. None of the victims with low socioeconomic status reported on day 0 compared with 30.9% (19–45) of bite victims with high status.

**Figure 4:**
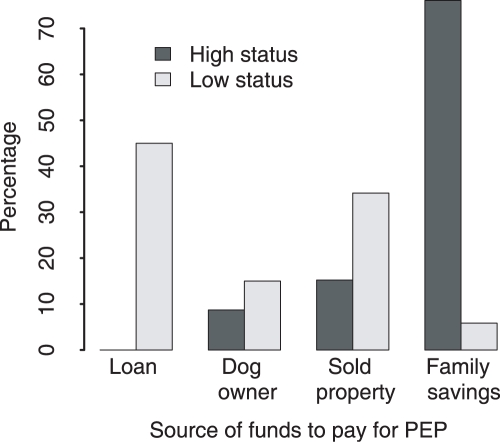
Means of obtaining funds to pay for PEP for rabies exposed individuals of high and low socioeconomic status.

Four major means of raising funds for PEP were reported: i) family savings; ii) borrowing money; iii) selling household properties and iv) payment by the owner of the rabid animal. Socioeconomic status had a significant impact on the source from which households obtained funds (p<0.0001). Households with higher socioeconomic status were more likely to use savings, whereas households with low socioeconomic status either obtained loans from relatives, friends and neighbors or depended on the owner of dogs which inflicted the bites to pay (Fig. 4).

Not all patients completed the PEP course, or adhered to the PEP schedule. Reasons given for not starting, completing or adhering to PEP regimes in the most commonly cited order were: i) unable to afford treatment; ii) no vaccine at the hospital; iii) the wound was small; iv) the dog owner would not pay; v) they were not aware the animal was rabid; vi) they were not aware of the danger of rabies; vii) medical staff did not advise PEP and viii) they thought they had received treatment but contact tracing revealed vaccination only against tetanus. Bite victims often quoted several reasons.

### Deaths

Twenty-eight deaths from suspected rabies were recorded during the five-year period in the two districts (Table 5), an average of 1.5/100,000 per year in Serengeti and 2.3 in Ngorongoro (Table 1). The odds of developing rabies following exposure were dramatically higher for those who did not receive PEP (odds ratio [OR] 17.33, 95% CI 6.39–60.83, p<0.0001). Accounting for the variation due to whether PEP was delivered or not, the odds of developing rabies were three-fold higher for children (<15 yrs) versus adults (OR 3.08, CI 1.10–11.04, p = 0.0498) and more than five-fold greater in Ngorongoro than Serengeti district (OR 6.12, CI 2.60–14.58, p<0.0001). A less powerful analysis that included only cases where PEP was not delivered showed the same patterns but only the effect of district was significant.

**Table 5:** Details of human rabies deaths.

#	Age (yrs)	Sex	District	Source of exposure	Bite site and details	Circumstances	Incubation period	Duration of clinical signs	Description of clinical signs	Reason for not receiving PEP
1	8	m	N	Dog	Shoulder, severe	While herding	3.5 m	3.5 d	Strange vocalisations	
2	5	m	N	Dog	Back, severe	While herding		4 d	Hypersalivation, strange vocalisations, ataxia	Referred to district hospital after symptoms began
3	7	m	N	Dog	Face	While herding	2 m	12 d	Strange vocalisations, hypersalivation, fever, headache, abnormal sleep	
4	14	m	N	Dog	Arm, severe	While herding	6 w			Received 4 doses
5	6	m	N	Dog	Face	While herding	1 m	12 d		Didn't realise dog was rabid
6	6	m	N	Dog					Aggression and agitation, bit mother	
7	13	m	N	Hyena	Hand, arms, severe	Bathing in the river	2 w	3 d		Not advised at dispensary where wounds treated
8	4	f	N	Dog			1 m		Fever, confusion, convulsions	
9	3	m	N	Dog						The wound had healed
10	16	m	N	Mongoose	Ankle, large septic wound	While herding	2 m		Aggression, anxiety, confusion	Didn't realise danger
11	5	f	N	Dog	Face	While herding	3 w	5 d	Strange vocalisations, hypersalivation	Advised to go to district hospital but it was too far
12	50	f	N	Cat	Hand and leg, severe	Bitten while in bed	1 m		Strange vocalisations, hypersalivation, dysphagia	
13	6	m	N	Honey badger	Head and hands	While herding with mother (who was also bitten)	1 m			
14	7	m	N	Dog					Fever	
15	7	m	S	Dog		While playing	31 d	5 d	Rashes, vomiting, fever, dysphagia, nervous, aggression, hypersalivation	Thought bitten by another healthy dog
16	8	f	S	Dog	Hands and wrist	Returning from school	33 d	6 d	Aggression, hypersensitive, vomiting, aerophobia, bit father	Dog owner lied about dog
17	45	m	S	Dog	Finger, deep punctures	Bitten by his own puppy while at home	29 d	4 d	Fever, shouting, confusion, dysphagia	No money
18	8	f	S	Dog	Head, hands, very severe	Returning from school	36 d	8 d	Fever, nervous, dysphagia	PEP given the following day
19	7	m	S	Dog	Face, hand, back, very severe	Returning from school	14 d	3 d	Convulsing, vomiting, anxiety, dysphagia	PEP given the following day
20	16	m	S	Dog	Arm and fingers	Was bitten at home	5 w			Attended hospital 10 days after bite
21	45	f	S	Dog			2 w			
22	15	m	S	Honey badger						
23	12	m	S	Dog	Hand and leg		9 m		Aggression, bit mother	No PEP remaining so went to local healer
24	11	m	S	Dog	Leg, small	While playing outside house	3 w	4 d	Headache, rash, aching, ataxia, nervous, hypersensitive, aerophobia	Not advised treatment because minor wound
25	5	m	S	Dog	Foot and ankle, small	Outside house	1 m	2 d	Ataxia, biting	
26	16	m	S	Dog	Leg		1 m	3 d	Strange vocalisations, biting till gums bled	Dog owner refused to pay
27	16	f	S	Cat			2 m		Aggression, hit mother	Health-worker said cat was bewitched
28	20	m	S	Dog	Leg	While hunting at night	3 m	4 d		Did not realise dog was rabid
29	70	f	S	Dog	Leg, severe	On way to village meeting	69 d	2 d	Paresthesia, strange vocalisations, agitation, pain	No money
30	45	f	S	Dog	Hand and face	Knocked down near house	53 d	1 d	Hypersalivation, hydrophobia	No money
31	40	f	S	Dog	Hand	Bitten at owners house	55 d		Hypersalivation, headache, unable to swallow	Given possibly fake injections at dispensary

Dog = domestic dog, Mongoose = white-tailed mongoose, m = month/s, w = week/s, d = day/s. Cases 29 to 31 occurred in 2007 and are not included in statistical analyses.

Three people who died from rabies received some PEP: two children in Serengeti district started PEP promptly (PEP was sought on the day of exposure, but delivered the following day because the medical facility was closed on weekends) and one teenager in Ngorongoro received the first dose of PEP several days after exposure and completed four doses before symptoms began. The vaccine in Ngorongoro district was tested and found to be viable. Vaccine was not available for testing in Serengeti but no other exposed patients died after receiving vaccine from the same batch. Moreover, the two children had severe injuries to the head, neck and spine, neither received immunoglobulins and the post-exposure regimen used was not WHO standard. One child developed symptoms shortly after receiving the second PEP dose and the second child died after completing the third dose. The remaining 25 cases did not receive any PEP, although at least 6 attended a medical facility promptly. Most rabies victims did not seek medical attention until after symptoms had begun, then in some instances the patient was taken to multiple medical centers in the hope of receiving a more positive prognosis. At least 5 cases (>17%) were initially diagnosed with cerebral malaria, but as symptoms progressed and when the history of a bite was discovered, the diagnosis was changed to rabies. Exposed individuals who developed rabies generally lived further from medical facilities than those who did not, although this was not statistically significant (p = 0.08). Risks of (and trauma from) human-to-human transmission are also not inconsequential; three rabies-infected individuals (>10%) bit a family member and a fourth hit her mother, apparently due to disease-induced changes including aggression. Additionally a twenty-year old woman died of tetanus following a suspected rabid dog bite. She developed symptoms of tetanus before completing her third dose of PEP. Because she was pregnant it was assumed that she must have been previously vaccinated against tetanus.

## Discussion

We investigated how risks of rabies exposure and onset of disease vary according to epidemiological and socioeconomic determinants and present evidence-based recommendations to reduce these risks in settings where canine rabies is endemic, addressing perspectives of both the health provider and patient [13].

Numbers of suspected rabies exposures varied considerably through time and across districts. The temporal variation was presumably due to the tendency of the disease to fluctuate on a timescale of approximately five years [4]. Assuming constant numbers of exposures per year may therefore be misleading if used as a basis for provisioning PEP. We suggest that exposure incidence, when used for indirect estimation of the burden of rabies, should be averaged over at least a five-year period because of inherent temporal variability. This study lasted five years, spanning one complete epidemic cycle and therefore the likely range of exposures through time. Our upper estimate of annual incidence of bite-injuries by suspect rabid animals in agro-pastoralist communities (100/100,000) is very close to previous estimates (104/100,000) [14]. However, vaccination of dog populations during the study substantially reduced the number of exposures and probably heightened awareness of the disease within study communities (several rabies-exposed individuals sought PEP after being interviewed). Our estimates therefore probably underestimate countrywide incidence, because mass dog vaccination campaigns are not routinely conducted across most of Tanzania. Heightened awareness may similarly explain our relatively low yet comparable estimates of annual rabies mortality (1.5 and 2.3/100,000 in Serengeti and Ngorongoro districts respectively) compared to previous estimates (4.9/100,00) [14].

The higher risk of exposure in the more populated areas was likely due to the higher incidence of rabies and longer duration of outbreaks (and less frequent fade-out) in larger domestic dog populations (dog density: ∼11.4/km^2^ in Serengeti district versus 4.2/km^2^ in Ngorongoro district, which is close to the critical threshold for persistence ∼4.5/km^2^) [15]. More abundant wild carnivores in Ngorongoro explains the high proportion of suspected rabies exposures caused by wild animals in the district [12]. Nevertheless, only the African 1b domestic dog associated rabies strain has been identified from the sequenced isolates (>50) in 9 species over the study area and evidence points to domestic dogs as the only population capable of rabies maintenance [16]. Control efforts should therefore be targeted towards domestic dog populations but education efforts should stress that the bite of any mammal can transmit rabies and should be treated promptly.

One of the greatest challenges for controlling canine rabies has been raising the priority of the disease. It is widely recognized that rabies is grossly under-reported even though it is notifiable and the lack of accurate figures has rendered rabies a low public health and veterinary priority. Previous attempts to quantify the burden of rabies have relied upon hospital records and have pointed out the need to verify their methods and conduct active case detection studies [6],[14],[17]. The validity of these indirect assessments is dependant upon key assumptions, such as the assumption that all rabies-exposed patients are recorded in hospital records. We show that at least 20% of all rabies exposures do not seek medical attention. Our estimates of rabies mortality are still comparable to model predictions, probably because the proportion of rabies-exposed individuals that received PEP, if medical attention was sought, was higher than during the previous study (0.86 versus 0.56) [14], though still unacceptably low. Thus, our contemporary data suggest indirect estimates of rabies-exposures and mortality based on well parameterized decision tree models are reasonable, but could be improved by accounting for the fact that not all bite victims seek PEP.

Our results highlight key aspects of health services that could be targeted to improve the treatment of patients reporting with animal-bite injuries. For instance, many bite victims had to travel to hospitals in neighboring districts (sometimes several) to obtain PEP, prolonging delays before PEP delivery, increasing the risk of disease and incurring considerable costs on victims and their families. Improved surveillance combined with timely reporting and centralized responses for vaccine distribution could prevent PEP shortages and reduce the need to travel to alternative clinics. Animal-bite injury records are an accurate indicator of rabies exposures (exposure status is not regularly recorded) and therefore have potential to be used as a surveillance tool, but to be of most value, records ought to be collated over catchment areas spanning several districts. The number of cases where patients reporting to medical facilities were misadvised is also unacceptable indicating that medical personnel require greater training in recognizing cases of rabies exposure and in judicious administration of appropriate PEP.

The risk and burden of rabies falls disproportionately on the most vulnerable sectors of society: children and particularly those in marginalized pastoralist populations. The high proportion of childhood rabies deaths, a well-documented statistic [18],[19], increases the disability-adjusted life years lost and therefore the burden of the disease [20]. Similarly those that live furthest from health facilities and are in lower socioeconomic classes undergo longer delays before receiving PEP which increases the risk of developing rabies. The high costs of PEP contribute to this problem, as many people must sell livestock or other possessions to raise funds. But many families spend even larger amounts of money trying to obtain treatment for a family member with clinical rabies than the total cost of preventative PEP, suggesting that the danger posed by the bite of a rabid animal is not fully appreciated. The substantially higher risk of developing rabies following exposure in Ngorongoro compared to Serengeti district cannot be explained by the probability of seeking medical attention. A plausible explanation is the adequacy of first aid delivered after a bite. Immediate washing of the wound considerably reduces the risk of disease progression [21], and may be practiced more in Serengeti than Ngorongoro. We do not have data to test this, but 7 of 14 deaths in Ngorongoro were children bitten whilst they were alone herding cattle, likely in remote areas, who probably did not administer appropriate first aid. Contact tracing uncovered many exposures and deaths not recorded in official sources, showing that the proportion of people exposed to rabies that seek PEP is unacceptably low. This results primarily from patients' lack of knowledge, or resources (or ability to mobilize them) suggesting that education to raise awareness about rabies prevention, wound management (particularly immediate flushing of the wound with any available liquid), and prompt PEP administration, could substantially reduce numbers of rabies deaths.

Zoonotic diseases are often neglected because the major burden falls within the health sector, yet the veterinary sector is usually responsible for their control. The two sectors typically operate independently and resources available to the medical sector are often much greater than those in veterinary departments. In reality rabies is a shared problem that can only be tackled by a multidisciplinary approach. Without laboratory confirmation and accurate diagnosis of animal rabies, public health authorities will not recognize rabies prevalence and without accurate information on human deaths and exposures from public health authorities the disease will not receive the attention it requires from the veterinary sector. One example that is a pervasive problem, evident in this and other studies [22], is the lack of diagnostic confirmation of human cases even though samples can be collected non-intrusively by supraorbital needle biopsy. Our results support previous findings that clinical diagnosis alone underestimates rabies incidence because of confusion with other neurological infections [8]. Nonetheless, the data we present provides a detailed picture of human rabies exposures and deaths during the last five years in a rural region of Tanzania; it leads to a number of practical recommendations for preventing future deaths ( Box 1) which should be valuable to medical practitioners and veterinarians alike. Misdiagnosis, incomplete understanding of how rabies is transmitted (for medical and veterinary workers and the general public), poverty and the lack of appropriate affordable treatment all result in needless human deaths. We highlight the practical problems that face people living in regions of endemic canine rabies and the tragically high prevalence of this disease which can be entirely controlled given sufficient political will.

###

**Box 1. Policy recommendations for reducing human deaths in canine rabies endemic regions**

In accordance with the Regional East African Community Health (REACH) initiative's mission to access, synthesize, package and communicate evidence required for policy and practice to improve population health and health equity (http://www.who.int/alliance-hpsr/evidenceinformed/reach/en/index.html) we provide recommendations for reducing human deaths from rabies following exposure.

1. Awareness needs to be raised about the importance of immediately washing animal-bite wounds and reporting rapidly to medical facilities for PEP (irrespective of the size and severity of injury).
2. Supply and distribution systems for PEP should be reviewed because shortages are frequent, regional disparities exist in prices and regimen, and treatment cannot always be accessed during evenings and weekends.
3. Mechanisms should be sought to reduce the price of PEP and enable early initiation of treatment for patients who may be unable to quickly access sufficient funds to pay for PEP (e.g. use of economical intradermal PEP regimens [23] for multiple patients who present simultaneously could be evaluated)
4. Improved training is needed for medical personnel to ensure awareness about the serious nature of rabies exposures and enable judicious decisions about PEP administration. Prophylaxis should be initiated immediately unless the patient is reporting more than ten days after exposure and is completely certain the biting animal is alive and healthy. Similarly PEP can be discontinued if the animal's good health can be established at subsequent hospital visits.
5. Collaborative (veterinary and medical) programs should be established to control and eliminate rabies from domestic dog populations and improve surveillance and diagnosis in both animal and human populations.

## Supporting Information

Alternative Language Abstract S1 Translation of the Abstract into Swahili by M. Kaare.
(0.01 MB PDF)
