# Supplementary material for: Rabies Exposures, Post-Exposure Prophylaxis and Deaths in a Region of Endemic Canine Rabies
Source: PLoS Negl Trop Dis. 2008 Nov 25;2(11):e339. doi: 10.1371/journal.pntd.0000339 (PMC2582685; doi:10.1371/journal.pntd.0000339)
Supplement: Alternative Language Abstract S1 — Translation of the Abstract into Swahili by M. Kaare. (0.01 MB PDF) [file pntd.0000339.s001.pdf]

Alternative Language Abstract S1 - Translation of the abstract into Swahili by M. Kaare.

Maambukizo ya kichaa cha mbwa, tiba baada ya maambukizo na vifo katika maeneo sugu ya ugonjwa wa kichaa cha mbwa

MUHTASARI

UTANGULIZI

Kichaa cha mbwa husababisha maelfu ya vifo vya binadamu kila mwaka japokuwa chanjo ifaayo dhidi ya ugonjwa huu kwa wanyama na binadamu inapatika. Dhumuni letu ilikuwa ni kutathmini vihatarishi vya maambukizo ya ugonjwa huu na kutambua kwa nini vifo vya binadamu vinavyosababishwa na kichaa cha mbwa vinaendelea kutokea katika maeneo yenye ugonjwa sugu

NJIA NA MATOKEO

Ufuatiliaji wa ana kwa ana ulitumika kukusanya takwimu za maambukizo ya ugonjwa wa kichaa cha mbwa, tiba itolewayo baada ya maambukizo na vifo katika maeneo mawili ya vivijini toka wilaya mbili zilizo kaskazini-magaharibi mwa Tanzania kuanzia mwaka 2002 hadi 2006. Takwimu za vihatarishi na mwenendo wa utafutaji na ukamilishaji wa tiba baada ya maambukizo ya kichaa cha mbwa zilikusanywa kwa kutumia dodoso katika kaya. Maambukizi yalitofautiana kutoka watu 6-141/100,000 kwa mwaka. Hatari ya kuambukizwa ilikuwa kubwa zaidi katika jamii zinazoendesha ufugaji na kilimo (na zenye mbwa wengi zaidi) kuliko katika jamii zinazoendesha ufugaji pekee (na zenye mbwa wachache) na pia kwa watoto zaidi kuliko watu wazima. Tiba baada ya maambukizo ilipunguza kwa kiwango kikubwa hatari ya kuugua kichaa cha mbwa (odds ratio [OR] 17.33, 95% confidence interval [CI] 6.39-60.83); pale ambapo tiba baada ya maambuki haikutolewa hatari ya kupta ugonjwa wa kichaa cha mbwa ilikuwa kubwa zaidi katika jamii za wafugaji kuliko katika jamii zinaondesha kilimo na ufugaji kwa pamoja (OR 6.12, 95% CI 2.60-14.58) na pia kwa watoto zaidi kuliko watu wazima. Uwezo mdogo wa kiuchumi na umbali toka vituo vya afya ambako chanjo hupatikana vilichangia uchelewashaji wa upatikanaji wa chanjo dhidi ya kichaa cha mbwa. Zaidi ya 20% ya watu walio ambukizwa kichaa cha mbwa hawakutafuta chanjo na taarifa zao hazikupatikana katika kumbukumbu rasmi na chini ya 65% ya watu walioambukizwa walipata chanjo baada ya maambukizo. Kumbukumbu ya taarifa za watu waliong'atwa na mbwa zilikuwa kiashiria sahihi cha kiwango cha maambukizo ya kichaa cha mbwa.

HITIMISHO

Ufahamu duni juu ya hatari itokanayo na ugonjwa wa kichaa cha mbwa na njia za kuuzia

ugonjwa huu, hususani kupata chanjo mara baada ya maambukizo na pia kuosha jeraha vilichangia kwa kiasi kikubwa katika usababishaji vifo kutoka na ugonjwa wa kichaa cha mbwa. Elimu juu ya ugonjwa huu hususani katika jamii masikini na zile zilizosahaulika, pia kwa wataalamu wa tiba ya binadamu na mifugo itasaidia kuzuia vifo vya uginjwa huu kwa siku za usoni.
